# Epidemiological, molecular characterization and antibiotic resistance of *Salmonella enterica* serovars isolated from chicken farms in Egypt

**DOI:** 10.1186/s13099-017-0157-1

**Published:** 2017-02-10

**Authors:** Hanem El-Sharkawy, Amin Tahoun, Abd El-Galiel A. El-Gohary, Moshira El-Abasy, Fares El-Khayat, Trudi Gillespie, Yukio Kitade, Hafez M. Hafez, Heinrich Neubauer, Hosny El-Adawy

**Affiliations:** 1 Department of Chemistry and Biomolecular Science, Faculty of Engineering, Gifu University, 1-1 Yanagido, Gifu, 501-1193 Japan; 2 Institute of Bacterial Infections and Zoonoses, Friedrich-Loeffler-Institut, Naumburger Str. 96a, 07743 Jena, Germany; 3 Faculty of Veterinary Medicine, Kafr Elsheikh University, Kafr El-Sheikh, 33516 Egypt; 4 CALM_live Imaging Facility, Centre for Inflammation Research, University of Edinburgh, Edinburgh, 47 EH16 4TJ UK; 5 Institute of Poultry Diseases, Free University Berlin, Berlin, Germany

**Keywords:** *Salmonella*, Broiler, Epidemiology, Antimicrobial, Integron

## Abstract

**Background:**

*Salmonella* is one of major causes of foodborne outbreaks globally. This study was conducted to estimate the prevalence, typing and antibiotic susceptibilities of *Salmonella enterica* serovars isolated from 41 broiler chicken farms located in Kafr El-Sheikh Province in Northern Egypt during 2014–2015. The clinical signs and mortalities were observed.

**Results:**

In total 615 clinical samples were collected from broiler flocks from different organs (liver, intestinal content and gall bladder). *Salmonella* infection was identified in 17 (41%) broiler chicken flocks and 67 *Salmonella* isolates were collected. Recovered isolates were serotyped as 58 (86.6%) *S. enterica* serovar Typhimurium, 6 (9%) *S. enterica* serovar Enteritidis and 3 (4.5%) were non-typable. The significant high mortality rate was observed only in 1-week-old chicks. *sop*E gene was detected in 92.5% of the isolates which indicating their ability to infect humans. All *S. enterica* serovar Enteritidis isolates were susceptible to all tested antimicrobials. The phenotypically resistant *S. enterica* serovar Typhimurium isolates against ampicillin, tetracycline, sulphamethoxazole and chloramphenicol were harbouring *Bla*TEM, (*tet*A and *tet*C), (*sul*1 and *sul*3) and (*cat*1 and *flo*R), respectively. The sensitivity rate of *S. enterica* serovar Typhimurium to gentamycin, trimethoprim/sulphamethoxazole and streptomycin were 100, 94.8, 89.7%, respectively. The silent streptomycin antimicrobial cassettes were detected in all *Salmonella* serovars. A class one integron (*dfr*A12, *orf*F and *aad*A2) was identified in three of *S. enterica* serovar Typhimurium strains.

**Conclusions:**

To the best of our knowledge, this study considered first report discussing the prevalence, genotyping, antibiotic susceptibility and public health significance of *S. enterica* serovars in broilers farms of different ages in Delta Egypt. Further studies are mandatory to verify the location of some resistance genes that are within or associated with the class one integron.

## Background

In spite of significant improvement in technology and hygienic practices at all stages of poultry production accompanied with advanced improvement in public sanitation, salmonellosis and *Salmonella* infections remains a persistent threat to human and animal health. In many countries high incidence of salmonellosis in man appears to be caused by infection derived from contaminated eggs, poultry meat and meat-products. The contaminated products cause disease as a result of inadequate cooking or cross contamination of working surfaces in kitchen environment [1–3].

The genus *Salmonella* of the family *Enterobacteriaceae* includes more than 3000 distinct serovars that have many host species and cause different diseases; most of which show little specificity for their host species [4–7]. The genus *Salmonella* can roughly be classified into three categories or groups: Group 1, highly host-adapted and invasive serovars: this group includes species restricted and invasive *Salmonella* such as *S.* Pullorum, *S.* Gallinarum in poultry and *S.* Typhi in humans. Group 2, non-host-adapted and invasive serovars: this group consists of approximately 10–20 serovars that are able to cause an invasive infection in poultry and may be capable of infecting humans. Currently, the most important serovars are *S.* Enteritidis, *S.* Typhimurium, *S.* Hadar, *S.* Heidelberg, *S.* Saintpaul *and S.* Infantis. Group 3, non-host-adapted and non-invasive serovars: most serovars of the genus *Salmonella* belong to this group and may cause disease in humans and other animals [8–14].

Although the acute enteritis caused by *Salmonella* species in humans is usually self-limiting, salmonellosis may be complicated especially in younger and older ages by severe systemic sequelae depending on serotype and on host-specific factors [15–17].

*Salmonella enterica* serovar Typhimurium and *S. enterica* serovar Enteritidis have been identified as the predominant serotypes present in Egyptian poultry farms [18].

*Salmonella enterica* serovar Enteritidis has been associated with disease in broiler breeding stock and can be transmitted vertically to their progeny [19]. Infection of adult chickens with *S. enterica* serovar Typhimurium is usually without clinical manifestation [20]. *S. enterica* serovar Enteritidis can inhabit the intestinal tract of several bird species such as chickens, turkeys and game birds and has the ability to survive outside of the host for over 1 year. *S. enterica* serovar Enteritidis infection in adult poultry is usually asymptomatic and infected bird will become a chronic carrier [21, 22]. In chickens up to 6 weeks of age *S. enterica* serovar Enteritidis may produce clinical symptoms including depression, disinclination to move, and diarrhoea, with high mortality especially in chicks less than 1 week of age [23], while older chicks may show uneven growth and stunting. Laying hens sometimes produce *S. enterica* serovar Enteritidis contaminated eggs leading to public health concerns [19]. The diseased birds may show lesions of pericarditis, perihepatitis and septicaemia. The mortality and morbidity vary and has been found to depend upon the dosage and phage type of the *S. enterica* serovar Enteritidis infection [24, 25].

Antimicrobial resistance is increasingly becoming an issue with salmonellosis infections in both animals and humans [26]. Understanding the key mechanisms involved in the evolution of antibiotics resistance in bacteria may aid scientific innovations aimed at controlling antimicrobial resistance [27, 28]. Bacteria can acquire resistance genes through mobile elements such as plasmids, which provide flexibility to host bacteria and help in the spread and distribution of these genes across diverse bacterial populations [29].

The inappropriate use of antibiotics in chicken farms in developing countries, including Egypt, is thought to be one of the main reasons for the increase in multidrug resistant bacteria [30]. These multidrug resistant bacteria including both *S. enterica* serovar Typhimurium, and *S. enterica* serovar Enteritidis that have the potential to infect humans and with a consequent failure of treatment can lead to systemic infection and death [31].

In this study, the incidence and antimicrobial resistance of *S. enterica* serovars Typhimurium and Enteritidis isolated from broiler chicken farms in Kafr El-Sheikh Province, Northern Egypt was reported. Determination of genes associated with antimicrobial resistance was investigated by examining the distribution of mobile integrons that carry the multidrug resistance cassettes within the genome of the isolated strains.

## Methods

### Sampling strategy and *Salmonella* isolation

This study was conducted in 41 broiler flocks located in Kafr El-Sheikh Province in Delta Egypt. Twenty flocks of 1-week-old birds and 21 flocks of 5-week-old birds were investigated. The observed clinical symptoms were observed and recorded (Table 1). Five living morbid birds from each flock were randomly selected and humanly sacrificed. At necropsy, sections of liver and intestinal wall plus contents were collected aseptically and processed for *Salmonella* isolation. From the same bird bile was aspirated from the gall bladder. Wetted cotton swabs in bacteriological transport media were used to collect samples from each specimen. Collected swabs and tissue samples were immediately frozen on ice and stored at −20 °C for further investigation within 5 h. Each tissue sample and swabs were inoculated in 10 ml selenite F broth (Oxoid, UK) and incubated at 37 °C overnight. A loopful of inoculated broth was streaked on selective *Salmonella Shigella* (SS) agar (Oxoid, UK) and incubated at 37 °C overnight. The suspected colony was sub-cultured on Xylose lysine deoxycholate (XLD) agar (Oxoid, UK) and on brilliant green (BG) agar (Oxoid, UK) and incubated at 37 °C for 16–18 h. The suspected colonies were collected for further biochemical identification using API 20E (BioMérieux, Marcy-l’Étoile, France).

**Table 1:** Flock description, signs, mortalities and *Salmonella* isolation rate from broiler chicken farms in the Kafr El-Sheikh Province Northern Egypt

Flock no.	No. of birds	Age/day	Clinical signs	Mortality, n (%)	Isolation results
1	10,000	1	Pasty diarrhea, blindness, lameness and high mortality	850 (8.5)	*S.* Enteritidis
2	10,000	2	Inappetence and respiratory manifestation	110 (1.1)	Negative
3	15,000	3	Pasty diarrhea, conjunctivitis, lowering in body weight and high mortalities	975 (6.5)	*S.* Enteritidis
4	15,000	4	Inappetence, ruffling feather and nervous signs	360 (2.4)	Negative
5	25,000	5	Lowering body rate and respiratory signs	550 (2.2)	Negative
6	2000	7	Pasty diarrhea, loss of appetite, ruffling feather and high mortalities	190 (9.5)	*S.* Typhimurium
7	5000	7	Decreased body weight, diarrhea, dehydration and high mortalities	415 (8.3)	*S.* Typhimurium
8	20,000	4	Decreased body weight	500 (2.5)	Negative
9	10,000	6	Whitish diarrhea, high mortalities, and decreased body weight	1160 (11.6)	*S.* Typhimurium
10	12,000	7	Inappetence, diarrhea and lowering body weight	540 (4.5)	Negative
11	25,000	7	Inability to move and nervous signs	850 (3.4)	Negative
12	30,000	5	Diarrhea, drop in feed intake and high mortalities	2610 (8.7)	*S.* Typhimurium
13	5000	4	Respiratory signs and decreased body weight	225 (4.5)	Negative
14	30,000	3	Inappetence, lowering growth rate	840 (2.8)	Negative
15	15,000	6	Whitish diarrhea, conjunctivitis and decreased body weight	945 (6.3)	*S.* Typhimurium
16	10,000	4	Diarrhea and decrease in body weight and respiratory signs	350 (3.5)	Negative
17	10,000	5	Inappetence, mortalities, lameness and diarrhea	550 (5.5)	*S.* Typhimurium
18	20,000	6	Decreased body weight and respiratory signs	640 (3.2)	Negative
19	12,000	5	Diarrhea, blindness and high mortality	648 (5.4)	*S.* Typhimurium
20	10,000	5	Respiratory and nervous signs	420 (4.2)	Negative
21	15,000	33	Inappetence and respiratory manifestation	375 (2.5)	*S.* Typhimurium
22	20,000	32	Decreased body weight	280 (1.4)	Negative
23	30,000	33	Mortalities	660 (2.2)	Negative
24	25,000	29	Nervous signs	625 (2.5)	Negative
25	15,000	34	Decreased body weight	345 (2.3)	*S.* Typhimurium
26	20,000	29	Decreased body weight	460 (2.3)	Negative
27	30,000	30	Decreased body weight	780 (2.6)	Negative
28	10,000	31	Respiratory signs and high mortality	330 (3.3)	*S.* Typhimurium
29	5000	28	Respiratory signs and mortalities	165 (3.3)	Negative
30	15,000	33	Inappetence and mortalities	480 (3.2)	Negative
31	20,000	33	Respiratory signs and high mortality	600 (3.0)	*S.* Typhimurium
32	20,000	32	Inappetence and respiratory manifestation	700 (3.5)	*S.* Typhimurium
33	10,000	28	Mortalities	200 (2.0)	Negative
34	20,000	29	Mortalities	480 (2.4)	Non typable *Salmonella* (three isolates)
35	10,000	33	Respiratory signs and mortalities	290 (2.9)	*S.* Typhimurium
36	5000	33	Lower body weight and respiratory signs	135 (2.7)	Negative
37	15,000	32	Nervous signs	345 (2.3)	Negative
38	20,000	33	Inappetence and mortalities	540 (2.7)	Negative
39	10,000	31	Respiratory signs and mortalities	320 (3.2)	*S.* Enteritidis
40	5000	29	Nervous manifestations and inappetence	140 (2.8)	Negative
41	25,000	35	Opisthosomas and ruffled feather	825 (3.3)	Negative

### Genomic DNA extraction and purification

The identified bacterial cultures were cultivated on SS agar and inoculated on Luria–Bertani (LB) broth (Oxoid, UK) and incubated at 37 °C overnight. The DNA was extracted from bacterial cultures on broth using Qiagen DNA extraction kit (Qiagen, UK) according to the manufacturer’s instructions.

### Molecular biological identification and differentiation of *Salmonella* serovars

In order to make a rapid and definite diagnosis of *Salmonella*, PCR was conducted using primers to detect the gene marker for *S. enterica inv*A [32], *sdf*I primers specific for detection of *S. enterica* serovars Enteritidis [33], and *Typ*h, *Sal* and *fliC* specific primers for serovar *S.* Typhimurium [34, 35] (Table 2).

**Table 2:** Primer sequences and their corresponding genes used for the detection of *S. enterica* serovar Enteritidis and *S. enterica* serovar Typhimurium

Gene	Oligonucleotide sequence (5′–3′)	Annealing (°C)	Amplicon size (bp)	Reference
*inv*A-F	GCT GCG CGC GAA CGG CGA AG	62	389	[32]
*inv*A-R	TCC CGG CAG AGT TCC CAT T			
*Sdf*I-F	TGTGTTTTATCTGATGCAAGAGG	58	293	[33]
*Sdf*I-R	CGTTCTTCTGGTACTTACGATGAC			
*Sdf*II-F	GCGAATATCATTCAGGATAAC	58	450	[33]
*Sdf*II-R	GCATGTCATACCGTTGTGGA			
*Sdf*III-F	GCTGACTCACACAGGAAATCG	58	350	[33]
*Sdf*III-R	TCTGATAAGACTGGGTTTCACT			
*Sef*A-F	GCC GTA CAC GAG CTT ATA GA	55	250	[33]
*Sef*A-R	ACC TAC AGG GGC ACA ATA AC			
*Sal fli*C-F	CCCCGCTTACAGGTGGACTAC	62	433	[35]
*Sal fli*C-R	AGCGGGTTTTCGGTGGTTGT			
*Sop*E-F	ACA CAC TTT CCA CGA GGA AGC G	55	398	[36]
*Sop*E-R	GGA TGC CTT CTG ATG TTG ACT GG			
*Typ*h-F	TTGTTCACTTTTTACCCCTGA A	55	401	[34]
*Typ*h-R	CCCTGACAGCCGTTAGATATT			

*inv*A positive strains were tested for the presence of the *sef*A gene, which encodes for SEF14 fimbriae that can be detected in *S. enterica* serovar Enteritidis strains and will also be present in the poultry-associated serotype *S.* Gallinarum.

In order to detect the zoonotic potential of our isolated strains of *S*. *enterica* serovar Enteritidis and *S*. *enterica* serovar Typhimurium we screened for the presence of the *sop*E gene [36].

The PCR reaction was geared to a previously described protocol for *Salmonella* [32–36]. Conserved forward and reverse primers (Eurofins, Japan) were used to generate the target amplicon (Table 1). The PCR cycling conditions were carried out as the following: initial denaturation at 94 °C for 5 min. Thirty cycles of amplification were run for 5 s, at 94 °C, 10 s at 68 °C and 20 s at 72 °C, with the final extension continuing at 72 °C for 7 min. Different annealing temperatures were used as described in Table 1. Five microliter aliquots of reaction mixture were electrophoresed through 1.5% agarose gels (Nippongene, Japan).

### Determination and sequencing of class 1 integrons

The class one integrons PCR fragments were purified from the agarose gel using Nucleospin Gel Extraction Kit (Macherey–Nagel, Germany) and sequenced (Genome centre—Gifu University, Japan). The sequencing results were analysed using BLAST webpage (http://blast.ncbi.nlm.nih.gov/Blast.cgi).

### Antimicrobial susceptibility testing

The antimicrobial susceptibility testing was performed using the Kirby–Bauer disc diffusion test [37] at the Clinical Veterinary Microbiology Laboratory of the Royal Dick School of Veterinary Study, University of Edinburgh. Briefly, one colony from the SS agar plate of each strain was picked up and streaked onto Mueller–Hinton blood agar (Oxoid, UK) and incubated at 37 °C overnight. Bacterial colonies were suspended in 0.9% NaCl to obtain a McFarland turbidity of 0.5 (Dr. Lange, photometer CADAS 30, Berlin, Germany) that containing about 1–2  ×  10^8^ colony forming units (CFU)/ml of *Escherichia coli* strain American Type Culture Collection (ATCC) 25922. Approximately, 300 μl of the saline suspension was spread onto the surface of a Mueller–Hinton agar plate (Oxoid, UK) using a sterile swab. The antimicrobial discs (Oxoid, UK) of six clinically used antibiotics that are used in the Egyptian poultry production (tetracycline 30 μg, ampicillin 10 μg, sulfamethoxazole/trimethoprim 25 μg, gentamicin 10 μg, streptomycin 25 μg and chloramphenicol 30 μg) were distributed onto the surface of the Mueller–Hinton agar plates using a Multi-disc dispenser (Oxoid, UK). The plates were incubated at 37 °C overnight. The diameters of the inhibited zones were measured using sliding callipers and interpreted using standard break points according to the method described by The European Committee on Antimicrobial Susceptibility Testing [38] (Table 3).

**Table 3:** Breakpoint values of each antimicrobial agent and phenotypic antimicrobial susceptibility profiles of 67 tested isolates used in this study according to EUCAST, 2015

Antimicrobial agents	Conc. (µg)	Diameter of inhibition zone (mm)			*S.* Typhimurium (58)			*S.* Enteritidis (6)			Non typable (3)		
		R	I	S	R	I	S	R	I	S	R	I	S
Ampicillin	10	≤13	14–16	≥17	58 (100%)	–	–	–	–	6 (100%)	3 (100%)	–	–
Chloramphenicol	30	≤12	13–17	≥18	58 (100%)	–	–	–	–	6 (100%)	3 (100%)	–	–
Gentamicin	10	≤12	13–15	≥16	–	–	58 (100%)	–	–	6 (100%)	–	3 (100%)	–
Streptomycin	25	≤11	12–14	≥15	–	6 (10.3%)	52 (89.7%)	–	–	6 (100%)	–	3 (100%)	–
Tetracycline	30	≤14	15–18	≥19	58 (100%)	–	–	–	–	6 (100%)	3 (100%)	–	–
Trimethoprim/sulphamethoxazole	25	≤10	11–15	≥16	3 (5.2%)	–	55 (94.8%)	–	–	6 (100%)	–	3 (100%)	–

*S* sensitive, *I* intermediate, *R* resistance

The gene associated with antibiotic resistance was tested in isolated *Salmonella* strains. Isolates were screened for the presence of 18 genes known to be associated with resistance to the seven tested antibiotics (Table 4).

**Table 4:** Primer sequences and their corresponding genes used for detection of antimicrobial resistant genes for *S. enterica* serovars

Gene	Primer	Nucleotide sequence (5–3)	Annealing (°C)	Amplicon size (bp)	Reference
*aad*A1	F	TATCAGAGGTAGTTGGCGTCAT	54	484	[62]
	R	GTTCCATAGCGTTAAGGTTTCATT			
*aad*A2	F	TGTTGGTTACTGTGGCCGTA	62	622	[62]
	R	GATCTCGCCTTTCACAAAGC			
*aad*B	F	GAGCGAAATCTGCCGCTCTGG	61	319	[62]
	R	CTGTTACAACGGACTGGCCGC			
*aac*C	F	GGCGCGATCAACGAATTTATCCGA	58	488	[28]
	R	CCATTCGATGCCGAAGGAAACGAT			
*bla*TEM	F	CATTTCCGTGTCGCCCTTAT	55	793	[62]
	R	TCCATAGTTGCCTGACTCCC			
*cat*1	F	CTT GTC GCC TTG CGT ATA AT	53	508	[27]
	R	ATC CCA ATG GCA TCG TAA AG			
*cat*2	F	CCGGATTGACCTGAATACCT	56	572	[62]
	R	TCACATACTGCATGATGAAC			
*dfr*I	F	GTGAAACTATCACTAATGGTAGCT	54	470	[62]
	R	ACCCTTTTGCCAGATTTGGTAACT			
*flo*R	F	AACCCGCCCTCTGGATCAAGTCAA	60	548	[62]
	R	CAAATCACGGGCCACGCTGTATC			
*str*A	F	AGCAGAGCGCGCCTTCGCTC	59	684	[62]
	R	CCAAAGCCCACTTCACCGAC			
*str*B	F	ATCGTCAAGGGATTGAAACC	49	509	[63]
	R	GGATCGTAGAACATATTGGC			
*sul*1	F	TCACCGAGGACTCCTTCTTC	60	316	[62]
	R	AATATCGGGATAGAGCGCAG			
*sul*2	F	CGGTCCGGCATCCAGCAATCC	64	441	[62]
	R	CGAGAGCCACGACCGCGCC			
*sul*3	F	GAGCAAGATTTTTGGAATCG	51	799	[63]
	R	CATCTGCAGCTAACCTAGGGCTTGGA			
*tet*A	F	GCTACATCCTGCTTGCCTTC	55	210	[64]
	R	CATAGATCGCCGTGAAGAGG			
*tet*B	F	TTGGTTAGGGGCAAGTTTTG	53	659	[64]
	R	GTAATGGGCCAATAACACCG			
*tet*C	F	CTTGAGAGCCTTCAACCCAG	56	418	[64]
	R	ATGGTCGTCATCTACCTGCC			
intI	F	5′GGCATCCAAGCAGCAAGC-3′	55	2000	[65]
	R	AAGCAGACTTGACCTGAT			

### Statistical analysis

The mortality rate associated with *Salmonella* infection and the rate of *S. enterica* serovar Typhimurium isolation from internal organs were analysed by the student t test [39].

## Results

### Clinical signs, mortality and incidence of *Salmonella* isolation from broiler flocks

Clinical symptoms of *Salmonella* infection observed in the 1-week-old broiler chicks included pasty diarrhea, inappetence, dehydration, growth retardation, blindness and lameness. The main gross lesions were hepatomegaly with necrotic foci, splenomegaly, pericarditis, panophthalmitis, and arthritis (Table 1).

In total 615 samples collected from intestine, liver and gall bladder from 41 broiler flocks, 67 (10.9%) *Salmonella* strains were isolated. In all, 45% of the sampled 1-week-old broiler flocks (9/20) and 38% of the screened 5-week-old broiler flocks (8/21) tested positive for *Salmonella* (Table 1).

The mean mortality rate (5.23% ± 2.85) of the 1-week-old flocks was significantly higher (P < 0.01) than the mean mortality rate (2.68% ± 0.52) in the 5-week-old flocks. When grouped by *Salmonella* infection status, the mortality rate observed in the 1-week-old birds was significantly higher (P < 0.001) in the *Salmonella* positive flocks (7.8% ± 2.07) compared to negative flocks (3.1% ± 0.45) (Table 1). While, there was no significant difference in mortality rate between the infected and non-infected 5-week-old flocks (P = 0.15, Table 1).

### Molecular biological identification of *Salmonella* serovars and public health significance

Both *S.* Enteritidis and *S.* Typhimurium serovars were isolated and identified from both the 1- and 5-week old sacrificed chicks (Table 5). Three of the collected 67 isolates were *Salmonella* positive but un-typable serovars.

**Table 5:** The rate of *S. enterica* serovars isolation from tissue organs collected from 41 broiler chicken flocks in Kafr El-Sheikh Province in Northern Egypt

Organs		Liver	Intestine	Gallbladder	Total
No. of collected samples		205	205	205	615
No. of isolates					
*S.* Enteritidis		2 (0.98%)	1 (0.49%)	3 (1.46%)	6 (0.98%)
* S.* Typhimurium		20 (9.76%)	8 (3.9%)	30 (14.63%)	58 (9.43%)
Un-typable *Salmonella*		0	3 (1.46%)	0	3 (0.49%)
Total		22 (10.74%)	12 (5.85%)	33 (16.09%)	67 (10.9%)

**Fig. 1:**
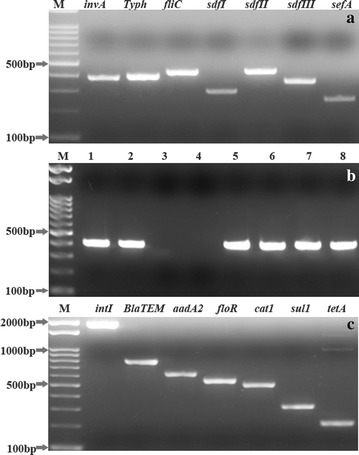
**a** Molecular biological identification of virulent gene associated with of *S. enterica serovars* Enteritidis and S. *enterica* serovar Typhimurium. *Lane M*, 100 bp DNA ladder. PCR amplification of *inv*A, *Typ*h, *fli*C, *sdf*I, *sdf*II*, sdf*III and *sef*A with expected amplicon size 389, 401, 433, ~293, ~450, ~350 and 250 bp, respectively. **b** Agarose gel electrophoresis of PCR amplifications of *sop*E gene in *S. enterica* serovars Enteritidis and *S. enterica* serovars Typhimurium. *Lane M*, 100 bp DNA ladder. *Lanes 1–2* and *5–8* were positive for the *sop*E with amplicon size 398 bp. **c** Molecular biological detection of antimicrobial resistant associated genes in *S. enterica* serovar Enteritidis and *S. enterica* serovar Typhimurium isolates. The amplified genes were *int*I integrons, *Bla*TEM, *aad*A2, *flo*R, *cat*1, *sul*1, and *tet*A genes with amplicon size 2000, 793, 622, 548, 508, 316 and 210 bp, respectively. *Lane M*, 100 bp DNA ladder

All 67 recovered isolates were harboured *inv*A gene (Fig. 1a). Out of 67 *inv*A positive *Salmonella* strains, 6 (9.0%) strains were positive for *sef*A, *sdf*I, *sdf*II and *sdf*III genes (Fig. 1a) indicating *S. enterica* serovar Enteritidis and 58 (86.6%) strains were positive for *Typ*h, *sdf*II and *fli*C marker (Fig. 1a) indicating *S. enterica* serovar Typhimurium. Three *Salmonella* strains (4.47%) were untypable and were positive for *inv*A and *sdf*II (Fig. 1a) (Table 5).

The isolation rate of *Salmonella* serovars from different organs were demonstrated in Table 5. Briefly, there was highly significant difference (P < 0.001) of *S. enterica* serovars Typhimurium isolated from the gallbladder (14.63%) and liver (9.76%) (P < 0.05) compared to those isolated from the intestine (3.9%) (Table 5). There was no significance difference (P = 0.28) between the isolation rate of *S. enterica* serovars Enteritidis form liver, intestine and gallbladder. The three un-typable serovars were found only at one farm and were isolated only from the intestinal samples (Table 5).

The *sop*E gene was amplified in 62 (92.5%) *Salmonella* isolates, indicating zoonotic and public health significance of isolated strains (Fig. 1b).

### Phenotypic and genotypic antimicrobial resistance

All *Salmonella* serovars isolated in this study were sensitive to gentamicin. Fifty-two (89.7%) *S. enterica* serovars Typhimurium isolates were susceptible to streptomycin, while six isolates (10.3%) were intermediate. Fifty-five (94.8%) *S. enterica* serovars Typhimurium isolates were sensitive to trimethoprim/sulphamethoxazole, while 3 (5.2%) isolates were resistant. All non-typable *Salmonella* strains were sensitive to trimethoprim/sulphamethoxazole and streptomycin. All *S. enterica* serovars Typhimurium and non-typable *Salmonella* strains isolated were resistant to ampicillin, chloramphenicol, and tetracycline. However, all *S. enterica serovars* Enteritidis isolates were sensitive to all tested antimicrobial agents (Table 3).

Ten of 18 screened resistance associated genes were amplified in the *S. enterica* serovars Typhimurium isolates (Table 6). All isolates harbourd *cat*1 associated with chloramphenicol resistance. While, 98.3, 96.6 and 94.8% of *S. enterica* serovars Typhimurium isolates were possessed *sul*3 (sulphamethoxazole resistance), *tet*C (tetracycline resistance) and *aad*A2 (streptomycin resistance), respectively. Moreover, 65.5, 84.5, 56.9, 62.1 and 79.3% of *S. enterica* serovars Typhimurium were harboured ampicillin (*Bla*TEM), tetracycline (*tet*A), sulphamethoxazole (*sul*1), streptomycin (*str*A) and chloramphenicol (*flo*R) resistance associated genes, respectively (Table 6; Fig. 1c). Eight of the 18 screened resistance genes were amplified in the *S. enterica* serovars Enteritidis isolates; these were tetracycline resistance *tet*A (50%), and *tet*C (33.3%); sulphamethoxazole resistance *sul*1 (16.7%); streptomycin resistance *aad*A1 (50%) and *str*A (33.3%); chloramphenicol resistance *cat*1 (33.3%) and *flo*R (16.7%). The un-typable *Salmonella* isolates were only positive for two genes; 100% for *tet*A (tetracycline resistance) and 33.3% for *cat*1 (chloramphenicol resistance). Only four of the screened genes *tet*B (tetracycline resistance), *sul*2 (sulfamethoxazole resistance) and *aad*B and *aac*C (gentamycin resistance) were not amplified in all screened isolates.

**Table 6:** Prevalence of antibiotic resistant associated genes detected in *S. enterica* serovars

Resistance markers	Prevalence of resistance genes in screened *Salmonella* isolates			Antimicrobial agent
	*S*. Typhimurium n = 58	*S*. Enteritidis n = 6	Non-typable n = 3	
*BlaTEM*	38	0	0	Ampicillin
*tetA*	49	3	3	Tetracycline
*tetB*	0	0	0	Tetracycline
*tetC*	56	2	0	Tetracycline
*sul1*	33	1	0	Sulphamethoxazole
*sul2*	0	0	0	Sulphamethoxazole
*sul3*	57	0	0	Sulphamethoxazole
*aadA1*	24	3	0	Streptomycin
*aadA2*	55	2	0	Streptomycin
*strA*	36	2	0	Streptomycin
*strB*	0	0	0	Streptomycin
*aadB*	0	0	0	Gentamycin
*aacC*	0	0	0	Gentamycin
*cat1*	58	2	1	Chloramphenicol
*cat2*	0	0	0	Chloramphenicol
*floR*	46	1	0	Chloramphenicol
*dfrI*	0	0	0	Trimethoprim
*intI*	3	0	0	Class I integron

The amplicons of *int*I integrons were identified with size of 2 kbp in three *S. enteric* serovar Typhimurium strains (Table 6). The sequencing data indicated that these integrons contain *dfr*A12-*orf*F-*aad*A2.

## Discussion

*Salmonella enterica* serovars Typhimurium is known to be able to cause high rates of mortality in early ages of broiler chickens [20]. The InvA protein is a putative inner membrane component of the *Salmonella* pathogenicity island 1 (SPI-1) type 3 secretion system (TTSS) [40]. It has been reported that *inv*A is present only in *Salmonella* species and therefore is used as a golden marker in genetic diagnosis of *Salmonella* species [35]. In this study 17 broiler flocks were positive and 67 *Salmonella* strains were isolated. The overall rate of incidence of *Salmonella* was (41%) in the screened broiler chicken flocks which was considerably higher than the infection rates that reported in the UK (10.7%), Lithuania (29%), Italy (20%), Netherlands (11%) and Germany (27.5% in chickens and 33.3% in turkeys) [41–45]. The higher infection rate found in this study compared to that of Abd El-Ghany et al. [18] shows the increased sensitivity of the use of the *inv*A gene marker for diagnosis compared to isolation through culture on specific agar.

Although the *S. enterica serovars* Enteritidis is closely related to other pathogenic *S. enteric* serovars, this serovar has some characteristics that appear to discriminate it from others serovars. As *S. enterica* is known to contain the *Salmonella* difference fragments (*sdf*), a group of chromosomally encoded genes, which to date are of unknown function. *sdf*I was reported by Agron et al. [33] to be found only in *S. enterica* serovars Enteritidis strains and considered to be a strong marker for this *Salmonella* serovar. *sdf*I was used as a target for phylotying of the serotype-specific *S. enterica serovars* Enteritidis. In this study, *sdf*I was present in 6 of the 67 *inv*A positive isolates. These *sdf*I positive strains were isolated from three of 41 screened farms. Our findings indicated that the *sdf*III gene marker was associated with the *sdf*I positive strains. Interestingly, *sdf*II was detected in all 67 strains isolated in this study of different serovars. This indicates that there is some degree of diversity within serovars that can be detected by the primers which in agreement with previous observation [33, 35].

Bacteria use the fimbriae in the adherence to one another and to the host cells and in some instance to inanimate objects. Sef14 fimbriae have been shown to consist of a repeating major subunit of the 14.3 kDa protein SefA, encoded for by the *sef*A gene and are required for macrophage uptake and survival in intraperitoneal infections [46]. The *sefA* gene is known to be specific to the poultry-associated *Salmonella* serotypes Gallinarum and Enteritidis. It is also detected in serotype Dublin, although this serotype is more commonly associated with cattle [47]. In the present study, *sef*A was detected in all isolates of *S. enterica* serovar Enteritidis. In the current study same six *S. enterica* serovar Enteritidis isolates positive for *sdf*I and *sdf*III markers were also positive for the *sef*A gene; these six strains came from three *Salmonella* infected chicken farms that were isolated from 41 screened farms.

In this study the *S. enteric* serovar Typhimurium serotype specific virulent flagella genes *Typ*h and *fliC* were used for phenotyping as recommended previously [34, 35]. Flagella are multi-functional organelles that play different roles in the biology of bacteria. The motility functions of flagella help bacteria to acquire nutrients, move away from toxic materials, and move to specific colonization sites within hosts and to disperse in the environment during the course of transmission between hosts [48]. The flagellum also primes the host immune system through activation of TLR5 receptors [49].

*sop*E is a translocated effector protein that plays an important part in the systemic phase of salmonellosis infection; *sop*E has been shown to be involved in actin cytoskeletal rearrangements and membrane ruffling [36]. As a virulence factor that is frequently transferred by bacteriophages, the *sop*E gene is encoded in the SPI-1, and has been identified in isolates involved in major epidemics; *sop*E has therefore been identified as playing a key role in the emergence of epidemic strains [50].

In study conducted by Rahman et al. [51] indicated that *sop*E gene appeared to be distributed and conserved among only a few serovars of *Salmonella* (Enteritidis, Gallinarum and Virchow) irrespective of their source of isolation and the presence of *sop*E gene in *Salmonella* provides an important pathogenic means to invade epithelial cells [51]. Moreover Prager et al. [52] identified *sop*E in all isolates of *S. enterica* serovar Enteritidis and carrying of *sop*E in *S*. Enteritidis may contribute to their epidemiological success [52]. In another study, all *Salmonella* Enteritidis isolated from human, chicken, and egg houses tested positive for *sopE* which may indicate its importance in pathogenesis [53].

In this study 92.5% of the *Salmonella* stains were harboured *sop*E gene that suggested that these strains could have zoonotic potential as previously reported [50–53].

There was a significant difference in mortality rate between *Salmonella* infected and non-infected flocks at the 1st week of life, however, there was no difference in mortality between *Salmonella* infected and non-infected flocks at the 5th week of age; a similar finding was previously reported [20, 23]. According to previous study, the results suggest that the age at infection plays an important role in the persistence of *S. enteritidis* infection in chickens and may cause severe infections and high mortality in young chickens [54]. Unfortunately, in this study we did not investigate other possible causes of mortality which may act as co-factors.

There was a higher rate of *Salmonella* isolation from the sampled internal organs, in the gall bladder and liver samples compared to the intestine samples indicating the ability of *Salmonella* to cause systemic infection which in agreement with previous study [55].

In this study All *S. enterica serovars* Typhimurium and non-typable *Salmonella* strains isolated in this study were resistance to ampicillin, chloramphenicol, and tetracycline.

All isolates were sensitive to gentamicin. The susceptibility of *S. enterica serovars* Typhimurium to streptomycin and trimethoprim/sulphamethoxazole were 89.7 and 94.8%, respectively. In addition 10.3% had intermediate sensitivity to streptomycin while all non-typable *Salmonella* strains were sensitive to trimethoprim/sulphamethoxazole and streptomycin. However, all *S. enterica serovars* Enteritidis isolates were sensitive to all tested antimicrobial agents. In contrast *Salmonella* isolates from South African chickens exhibited resistance to tetracycline (93%), trimethoprim–sulfamethoxazole (84%), gentamicin (48%), ampicillin (47%), chloramphenicol (31%), and streptomycin (12%) [56].

Most of the phenotypically antibiotic resistance isolates were positive for some of the antibiotic resistance marker genes for each of the screened antibiotics.

The *bla*TEM gene was detected only in 65.5% of ampicillin resistant *S. enterica* serovar Typhimurium isolates. All of the isolated strains of *S. enterica* serovar Enteritidis were susceptible to ampicillin and were negative for *bla*TEM. The three non-typable *Salmonella* strains showed phenotypical resistance to ampicillin without harbouring the *bla*TEM gene, indicating that these strains possess another ampicillin resistance mechanism.

In this study, tetracycline resistance in the *S. enterica* serovar Typhimurium isolates correlated with the presence of *tet*C (96.6%), and *tet*A (84.5%). All tested strains were negative for *tet*B codon. *tet*A codon was also found in all of the non-typable *Salmonella* strains. All *S. enterica* serovar Enteritidis were sensitive to tetracycline. However, two of the strains were harboured both *tet*C and *tet*A determinants and one strain was harbouring *tet*A determinant. These cassettes were silent in this serotype strain in vitro, however, they may turn on in vivo.

All of the *S. enterica* serovar Enteritidis and non-typable *Salmonella* strains were sensitive to trimethoprim–sulphamethoxazole and all these strains were negative for the *dfr*1 codon and did not possess integron that contains *dfr*A12 trimethoprim resistance cassette. Although one strain of *S. enterica* serovar Enteritidis carried *sul*1 gene but not possessed any trimethoprim genes. All *S. enterica* serovar Typhimurium isolates were sensitive to trimethoprim–sulphamethoxazole despite 98% of isolates being positive for *sul*3 and 57% being positive for *sul*1, both of which confer sulphamethoxazole resistance. Interestingly, the three *S*. Typhimurium strains that were resistant to trimethoprim–sulphamethoxazole were found to harbour the 2 kp integron that contains the *dfr*A12 trimethoprim resistant marker.

All of the *Salmonella* isolates were sensitive to the streptomycin despite the presence of streptomycin modifying enzyme gene cassettes (*aad*A1, *aad*A2 and *str*A). This suggests that some of the antimicrobial resistance genes are silent in bacteria in vitro; however, these silent genes can spread to other bacteria or turn on in vivo, especially under antimicrobial pressure which in agreement with previous reports [31, 57].

The *cat1* gene, encoding chloramphenicol acetyltransferase, was identified in all resistant strains. In *S. enterica* serovar Typhimurium, the *cat*2 gene was not found in any of the tested strains. The *flo*R gene which also confers chloramphenicol resistance was detected in 80% of *S. enterica* serovar Typhimurium strains. One of the non-typable *Salmonella* strains carried the *cat*1 gene but the other two isolates did not possess *cat*1*, cat*2 or *flo*R gene indicating that these two strains harbour another chloramphenicol resistance mechanism. Of six *S. enterica* serovar Enteritidis strains, one strain possessed both, *cat1* and, *flo*R, and one strain harboured only the *cat*1 gene, however, phenotypically they were all sensitive to chloramphenicol indicating that this resistant cassette is silent in vitro in this *Salmonella* serovar.

Multiple drug resistance genes have been found to be clustered on individual mobile elements, which mean that multi-resistance can be readily transferred and increase the multi-drug resistant bacterial population as reported previously [58].

Gene cassettes are a major source of the resistance genes found in clinical, commensal, and environmental isolates of bacteria that are resistant to antibiotics [59, 60]. Most commonly, they are found in association with class 1 or class 2 integrons [61].

In this study, a class one integron in three *S. enterica* serovar Typhimurium strains with size of 2 kb was identified. The sequencing data indicated that these integrons contained *dfr*A12-*orf*F-*aad*A2. The presence of the *dfr*A12-*orf*F-*aad*A2 open reading frames revealed the basis for the streptomycin and trimethoprim/sulphamethoxazole resistance seen in these strains. It also provides an indication of the mapping distribution of antibiotic resistance alleles in this region of the *Salmonella* genome/chromosome.

In this study the higher infection rate in the investigated flocks may regarding to low biosecurity and hygienic measures inside these farms and easily to spread the infection through different reservoirs and the workers in the farms.

The screening of antimicrobial resistance in the *Salmonella* strains isolated in this study provides evidence for confirming the mechanisms employed by *S. enterica serovars* to resist cluster antibiotics used for treatment of broiler chicken in Egypt. Future work, in this regard, should address if allele distribution in chicken and human *Salmonella* isolates from the same region share the same resistance mechanisms in order to highlight potential horizontal gene transfer by this zoonotic organism and the origin of antimicrobial resistance in human isolates. Finally, we believe that this is the first report of the presence of a class one integron in the *S. enterica* serovar Typhimurium serotype together with the verification of the location of some resistance genes that are within or associated with the class one integron.
